# Hampa: Solver-Aided Recency-Aware Replication

**DOI:** 10.1007/978-3-030-53288-8_16

**Published:** 2020-06-13

**Authors:** Xiao Li, Farzin Houshmand, Mohsen Lesani

**Affiliations:** 8 Microsoft Research Lab, Redmond, WA USA; 9 University of Southern California, Los Angeles, CA USA; University of California, Riverside, USA

## Abstract

Replication is a common technique to build reliable and scalable systems. Traditional strong consistency maintains the same total order of operations across replicas. This total order is the source of multiple desirable consistency properties: integrity, convergence and recency. However, maintaining the total order has proven to inhibit availability and performance. Weaker notions exhibit responsiveness and scalability; however, they forfeit the total order and hence its favorable properties. This project revives these properties with as little coordination as possible. It presents a tool called $\textsc {Hampa}$ that given a sequential object with the declaration of its integrity and recency requirements, automatically synthesizes a correct-by-construction replicated object that simultaneously guarantees the three properties. It features a relational object specification language and a syntax-directed analysis that infers optimum staleness bounds. Further, it defines coordination-avoidance conditions and the operational semantics of replicated systems that provably guarantees the three properties. It characterizes the computational power and presents a protocol for recency-aware objects. $\textsc {Hampa}$ uses automatic solvers statically and embeds them in the runtime to dynamically decide the validity of coordination-avoidance conditions. The experiments show that recency-aware objects reduce coordination and response time.

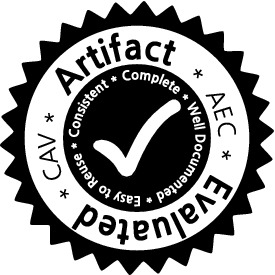



## Introduction

Replicated objects [12, 13, 23, 32, 45] are pervasively used for fault-tolerance, availability, responsiveness and scalability. They are used in diverse application areas [14, 20–22, 37, 39, 40, 50, 53] including embedded controllers, online services and game engines. However, coordinating the replicas has proven to be challenging. Strongly consistent replication, provided by consensus protocols such as Viewstamp [42], Paxos [34] and Raft [44], guarantees the same total order of operations across replicas. The total order simultaneously provides a hoard of favorable properties: integrity, convergence and recency. Replicas converge to the same state as the result of the same sequence of operations. Further, a propagated operation executes in the same state as the originating replica. Therefore, if an operation preserves the integrity properties [8] at the originating replica, it will certainly preserve them in the other replicas as well. In addition, the lock-step execution keeps the replicas recent: an operations executes in all replicas before the next. Thus, replicas can be stale by at most one operation.

However, strong consistency may not be available and responsive during network failures or offline use. Further, its scalability is limited. The trade-off between strong consistency of replicated objects, and their availability and responsiveness is a famous dilemma [1, 3, 26–28]. Therefore, system designers opted for weaker notions of consistency such as eventual [4, 15, 17, 19, 24, 25, 48, 52] and causal [2, 13, 33] consistency that can provide availability, responsiveness and scalability but lose the same total order of operations. Several projects [16, 49, 51] provide programming interfaces for weak consistency notions. Unfortunately, the large collection of subtle weak consistency notions is unintuitive to users. If the chosen notion is too weak, it can affect correctness, and if it is too strong, it may degrade scalability.

Therefore, researchers have recently provided high-level abstractions to shield the user from low-level complexities of weak consistency. These projects seem to be the steps towards reviving the same three pillars of consistency, i.e. integrity, convergence and recency, with as little coordination [7, 35, 47] as possible. CRDTs [48] revived convergence. If an object satisfies a few algebraic properties, its replication can enjoy convergence even on top of eventual consistency. However, the replicas can experience states that violate the integrity properties. Therefore, follow-up projects revived the integrity property. CISE [29] and Soteria [41] present proof techniques to verify the integrity properties of a replicated object. Sieve [36], Indigo [10] and Hamsaz [30] translate the given high-level integrity properties to hybrid models. However, they are oblivious to state recency. The operations are eventually delivered to all replicas, however, they may be arbitrarily delayed. Some updates may be delivered too late and expose the clients to stale data. On the other hand, at the expense of more communication, some updates may be immediately sent and delivered. However, applications may prefer to obtain more scalability and energy efficiency in return for bounded staleness. In fact, many applications such as ticketing, distributed sensors and network accounting can work with fairly recent data. Previous work such as TACT [55], TRAPP [43], FRACT [59], and PBS [9] considered staleness but did not address integrity and communication minimization. Further, they did not provide automatic analysis, decision and synthesis. In addition to convergence and integrity, this project, $\textsc {Hampa}$, revives recency. Given a sequential object with the declaration of its integrity properties and recency requirements for its methods, it automatically synthesizes a correct-by-construction replicated object that guarantees integrity, convergence and recency while avoiding unnecessary coordination.

To capture object specifications from the user, we present a relational language and its denotational semantics. The language provides a complete set of relational operators to define the object methods and integrity properties, and allows the user to declare recency requirements for the return value of each method. Given a principled object specification, we present a syntax-directed analysis that infers optimum staleness bounds for each element of the state.

We present the conditions required to simultaneously preserve the three properties: convergence, integrity and recency. These conditions are used to define a novel operational semantics of replicated objects that provably preserve convergence, integrity and the inferred staleness bound. We observe that recency-awareness not only guarantees a limit on the staleness, but also allows buffering of calls and reduces the coordination required to preserve integrity.

We characterize the computational power of recency-aware replicated objects. We show that recency-aware objects have the same power as the perfect failure detector. We present a novel protocol for recency-aware replicated objects that implements the semantics. We use off-the-shelve SMT solvers both statically and embed them at runtime to decide the validity of coordination-avoidance conditions. We present a tool called $\textsc {Hampa}$ that given an object definition, analyzes the object and instantiates the protocol to synthesize replicated objects. Our experiments with the synthesized objects show that the staleness bound has an inverse relationship with the coordination and response time.

In summary, this paper presents the following contributions: (1) A relational object specification language that captures integrity and recency declarations, and its denotational semantics (Sect.  2). (2) The coordination conditions and the operational semantics of replicated systems that simultaneously preserve convergence, integrity and recency (Sects.  3 and 4). (3) A syntax-directed analysis that infers optimum staleness bounds for each element of the state (Sect.  5). (4) The characterization of the computational power and a protocol for recency-aware replicated objects, (Sect. 6). (5) The $\textsc {Hampa}$ replicated object synthesis tool and its experimental results (Sect. 7). All the proofs are available in the appendix [5].

## Recency-Aware Relational Object Language

**Fig. 1.**
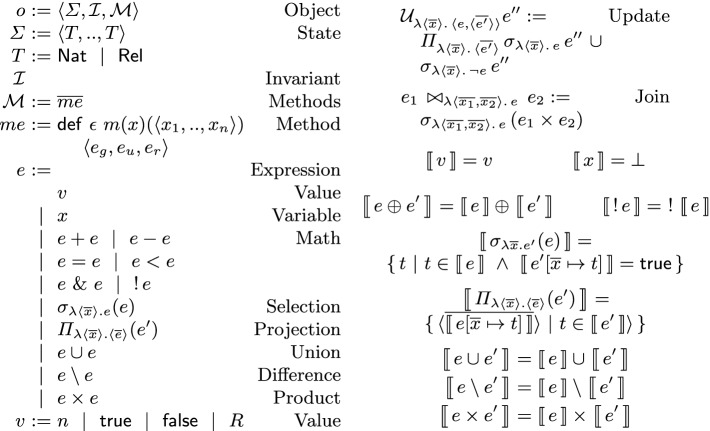
Syntax and semantics of the specification language

**Language.** Figure 1 shows our core relational language for object specification. An object is a record $\langle \varSigma , \mathcal {I}, \mathcal {M}\rangle$ that includes a state type $\varSigma$, an invariant $\mathcal {I}$ on the state, and a set of methods $\mathcal {M}$. The state can be a tuple of natural number $\mathsf {Nat}$ and relation $\mathsf {Rel}$ types. The invariant $\mathcal {I}$ is a boolean function on the state. A method *m* is a function from the parameter *x* and the pre-state $\langle x_1, .., x_n\rangle$ to a record of $\langle e_g, e_u, e_r\rangle$. The guard $e_g$ is a boolean expression that captures the semantic preconditions of *m* such as conditions on the arguments. The expressions $e_u$ and $e_r$ are for the post-state and the return value. We use $\textsf {guard}$, $\textsf {update}$ and $\textsf {retv}$ as functions that extract elements of this record. For each method, the user declares an integer as the staleness bound $\epsilon$ for its return value. A method call *c* is a method applied to its argument i.e. it is a function from the current state to a record of $\langle e_g, e_u, e_r\rangle$.

An expression *e* is either a value *v* (that can be either a number *n* or a relation *R*), a variable denoted by *x*, an application of the operators $\{ +, -, =, <,  \& , ! \}$ to operand expressions where & is the conjunction and and ! is the negation operator, a selection $\sigma _{\lambda \langle \overline{x}\rangle . e}(e')$ that binds the attributes of each element of the relation $e'$ to the variables $\overline{x}$ and returns the elements that satisfy the condition *e*, a projection $\varPi _{\lambda \langle \overline{x}\rangle . \langle \overline{e}\rangle }(e')$ that for each element of the relation $e'$, binds its attributes to the variables $\overline{x}$ and calculates a tuple of elements $\langle \overline{e}\rangle$ and returns the set of resulting tuples, a union $e \cup e'$ that results in a relation with elements of both of the relations *e* and $e'$, a difference $e \setminus e'$ that results in a relation with the elements in the relation *e* that are not in the relation $e'$, and the Cartesian product $e \times e'$ that results in a relation with pair elements where the first and second elements are in the relations *e* and $e'$ respectively. The language supports a complete set of relational operators: any relational algebra expression can be expressed by a combination of them. Selection ($\sigma$), projection ($\pi$), union ($\cup$), difference ($\setminus$), product ($\times$) and renaming ($\rho$) are a complete set of operators. We note that since the language uses functions with argument names, a renaming operator is unnecessary. The update and join operations are defined as a syntactic sugar. The update operation $\mathcal {U}_{\lambda \langle \overline{x}\rangle . \, \langle e, \langle \overline{e'}\rangle \rangle } e''$ returns a relation that updates each element of $e''$ that satisfies the condition *e* to the tuple $\langle \overline{e'}\rangle$. The join $e_1 \, \bowtie _{\lambda \langle \overline{x_1}, \overline{x_2}\rangle . \, e} \, e_2$ results in pairs of elements of $e_1$ and $e_2$ that satisfy the condition *e*.

**Semantics.** Figure 1 presents a denotational semantics for expressions. The semantics for values, variables, and binary and unary operations is standard. The semantics of the selection expression $\sigma _{\lambda \langle \overline{x}\rangle . e'}(e)$ is the set of tuples *t* in the semantics of *e* such that substitution of the attributes $\overline{x}$ in $e'$ with their corresponding values in *t* evaluates to true. The semantics of the projection expression $\varPi _{\lambda \langle \overline{x}\rangle . \langle \overline{e}\rangle }(e')$ is a set of tuples, one per each tuple *t* in the semantics of $e'$: a tuple resulted from substituting $\overline{x}$ with *t* in the expressions $\overline{e}$ and evaluating them. The semantics of union, difference and product are standard from the set theory. We define the difference $\varDelta$ between two values as follows: the difference between two natural numbers is the absolute value of their subtraction i.e. $\varDelta (n, n') = |n - n'|$; the difference of two relations is the size of their symmetric difference i.e. $\varDelta (R, R') = |R \setminus R'| + |R' \setminus R|$. We use delta $\delta$ to represents the staleness of a value that is the difference between the value and its target value. The delta for a completely recent (or exact) value is zero. For a call *c*, the weight $\textsf {weight}(c)$ is a bound on the difference that the execution of *c* can make on the state of the object. In other words, for every call *c*, we have $\forall \sigma . \ \text{ Let } \langle \_, \sigma ', \_ \rangle := c(\sigma ) \text{ in } \varDelta (\sigma ', \sigma ) < \textsf {weight}(c)$.

**Fig. 2.**
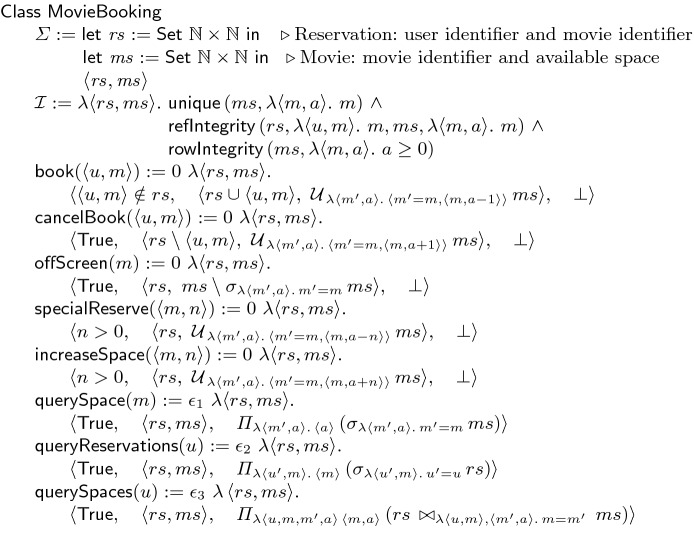
Movie booking use-case

**Running Use-Case.** Figure 2 shows the movie booking use-case. The state of the object is the two relations reservation $rs$ and movie $ms$. The reservation relation $rs$ stores the movies that the users have booked; it is the pairs of users *u* and movies *m*. The movie relation $ms$ stores the number of available spaces for each movie; it is the pairs of movies *m* and spaces *a*. The integrity property $\mathcal {I}$ is a conjunction of three conditions: (1) The movie in $ms$ should be unique. (2) The referential integrity requires that every movie in $rs$ exists in $ms$. (3) The number of available spaces for every movie should be non-negative. The object provides five update methods and three query methods. Given a user *u* and a movie *m*, the method $\textsf {book}$ adds the pair to $rs$ and decrements the available spaces for *m* in $ms$. Similarly, the method $\textsf {cancelBook}$ removes a reservation and increments available spaces. Given a movie *m*, the method $\textsf {offScreen}$ removes the corresponding tuple from $ms$. Given a movie *m* and a number *n*, the method $\textsf {specialReserve}$ subtracts *n* from the available spaces for *m* in $ms$. The dual method $\textsf {increaseSpace}$ adds *n* to the spaces for *m*. Given a movie *m*, the method $\textsf {querySpace}$ returns the number of available spaces for *m*. The method $\textsf {queryReservations}$ returns the set of movies that the given user has booked. Given a user *u*, the method $\textsf {querySpaces}$ returns the pairs of movies and their available spaces for the movies that *u* has booked. The staleness bound for the update methods is specified as 0. The returned none constant $\bot$ is always exact. The bound values $\epsilon _1$, $\epsilon _2$ and $\epsilon _3$ of the query methods represent the number of tuples that are different between the current state and the pending stable state of the result relation.

**Fig. 3.**
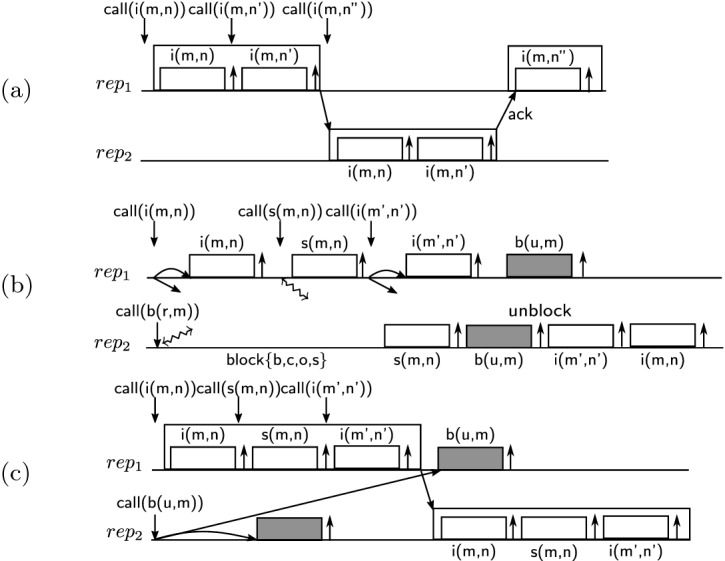
(a) Buffering and coordination. Example execution (b) without and (c) with recency. $\downarrow$: request, $\uparrow$: indication, $\leftrightsquigarrow$: synchronization

To reduce communication, certain calls can be executed locally and buffered, and the buffer can be communicated to other replicas later. As an example, in Fig. 3(a), the first two calls to the method $\textsf {increaseSpace}$ do not exceed the staleness bound for $ms$ and can be buffered. However, the third call exceeds the bound and cannot be added to the buffer. Therefore, the buffer is flushed to other replicas and the third call is blocked until an acknowledgement for the delivery of the buffer is received. All the calls of the buffer can be sent in a single message and the acknowledgement for them can be sent in a single message as well.

Let us now consider the interaction of buffering with coordination. We will see that buffering (staleness) interestingly reduces the coordination required for the conflicts. (We will define conflicting calls that should be synchronized later in Sect. 3.) Fig. 3(b) and (c) show the same execution without and with buffering respectively. In Fig. 3(b), the first replica $$ rep _1$$ executes the sequence of calls $$\textsf {increaseSpace}$$, $$\textsf {specialReserve}$$ and $$\textsf {increaseSpace}$$. The method $$\textsf {increaseSpace}$$ does not conflict with any other method; therefore, calls to it are simply broadcast. The method $$\textsf {specialReserve}$$ conflicts with itself and the method $$\textsf {book}$$; therefore, the call to it goes through synchronization. The second replica $$ rep _2$$ calls $$\textsf {book}$$ that conflicts with four other methods. Hence, it should synchronize. (The synchronization reaches to other replicas, blocks calling the four methods, and propagates previous calls to those methods.) In this example, the conflicting $$\textsf {specialReserve}$$ call in $$ rep _1$$ should be propagated to $$ rep _2$$ before the $$\textsf {book}$$ call can be executed.

In Fig. 3(c), the recency bound allows the three calls of $$ rep _1$$ to be buffered. Replicas use SMT solvers at runtime to check the validity of three properties for the buffers: all-$$\mathcal {S}$$-commutativity, invariant-sufficiency and let-$$\mathcal {P}$$-R-commutativity that we will formally define in Sect. 3. In this example, the buffer is invariant-sufficient if the number of spaces that the call $$\textsf {specialReserve}$$ decrements is less than the number that the $$\textsf {increaseSpace}$$ calls increment. Therefore, the buffer can be sent to other replicas without any additional synchronization; the invariant in the pre-state is sufficient for the invariant in its post-state. We note that the call $$\textsf {specialReserve}$$ that previously went through synchronization does not need any synchronization inside the buffer. Further, the let-$$\mathcal {P}$$-R-commutativity property of the buffer guarantees that the $$\textsf {book}$$ call will preserve the integrity after the buffer. Thus, the synchronization of the $$\textsf {book}$$ call that previously waited for the $$\textsf {specialReserve}$$ call does not need to wait anymore.

## Coordination Conditions

In this section, we present the coordination conditions for replicated objects that preserve the three properties: convergence, integrity and recency. The state of the given sequential object is replicated across replicas. Clients can request method calls at every replica, and replicas coordinate the calls. *Convergence* is the safety property that when all pending updates are processed, the replicas converge to the same state. *Integrity* is the safety property that every method call is executed only on a state where the guard of the method and the invariant are satisfied. *Recency* is the safety property that bounds the difference between the state of a replica and its impending state after the pending calls are applied.

The state of each replica is initialized to the same state $\sigma _0$ that satisfies the invariant $\mathcal {I}$. The replica that accepts the request for a call from the user is called the originating replica of the call. We uniquely identify requests by identifiers *r*. We use the two maps $\textsf {call}$ and $\textsf {orig}$ that map request identifiers to the method call and originating replica respectively. The execution history of a replica is modeled as a permutation of a set of request identifiers. An execution $\textsf {x}$ of a set of requests *R* is a bijective from positions $[0..|R|-1]$ to *R*. We denote the range of $\textsf {x}$ as $R(\textsf {x})$. An execution $\textsf {x}$ of *R* defines the total order $\prec _\textsf {x}$ on *R*: A request *r* precedes another request $r'$ in an execution $\textsf {x}$ written as $r \prec _\textsf {x}r'$ iff $\textsf {x}^{-1}(r) < \textsf {x}^{-1}(r')$. A replicated execution $\textsf {xs}$ is a function from replicas $\mathcal {N}$ to executions. The post-state of each call at a replica is the result of applying the call to its pre-state.

We first revisit the coordination conditions for convergence and integrity [30], and then present coordination conditions for recency and their impact on the prior conditions.

**Convergence.** A replicated execution is convergent if the state of the replicas is the same after all the calls are propagated. Out of order delivery of method calls at different replicas can lead to divergence of their states. Method calls such as special reservation $\textsf {specialReserve}$ and increasing space $\textsf {increaseSpace}$ result in the same state if their order of execution is swapped. However, the resulting state of the two method calls $\textsf {book}$ and $\textsf {cancelBook}$ is dependent on their execution order. Therefore, they should synchronize.

### Definition 1 (State-Commutativity and State-Conflict)

Two method calls $c_1$ and $c_2$ $\mathcal {S}$-commute, written as $c_1 \leftrightarrows _\mathcal {S}c_2$ iff for every state $\sigma$, $\mathsf{{update}}(c_2)(\mathsf{{update}}(c_1)(\sigma ))$ $=$ $\mathsf{{update}}(c_1)(\mathsf{{update}}(c_2)(\sigma ))$. Otherwise, they $\mathcal {S}$-conflict, written as $c_1 \bowtie _\mathcal {S}c_2$.

**Integrity.** The body of each method relies on the invariant in the pre-state. Further, methods have explicit guards that declare their pre-conditions. We say that a method call enjoys integrity at a state if the invariant and the guard of the method hold in that state.

### Definition 2 (Integrity)

A method call *c* enjoys integrity in a state $\sigma$, written as $\textsf {integrity}(\sigma , c)$, iff $\textsf {guard}(c)(\sigma )$ and $\mathcal {I}(\sigma )$.

Method calls should be executed only in states that they have integrity in. The integrity condition is simply lifted to executions and replicated executions: An execution enjoys integrity iff every request in it enjoys integrity.

### Definition 3 (Permissibility)

A method call *c* is permissible in a state $\sigma$, written as $\mathcal {P}(\sigma , c)$, iff $\textsf {guard}(c)(\sigma )$ and $\mathcal {I}(\mathsf{{update}}(c)(\sigma ))$.

In contrast to integrity that requires the invariant to hold in the pre-state, permissibility requires it to hold in the post-state. The post-state of a call is the pre-state of the next call in a replica. Further, the initial state is assumed to satisfy the invariant. Therefore, if every call is permissible in its pre-state, then every call enjoys integrity. By induction, permissibility leads to integrity.

To execute a method call, we check that it is permissible at its originating replica. Thus, we say that each method call is *locally permissible*. Otherwise, the call is aborted or delayed. Still, if the call is simply broadcast, it is not necessarily permissible when it arrives at other replicas. Some calls need coordination.

**Conflict.** There are calls such as $\textsf {increaseSpace}$ that are always permissible as far as they are applied to a state that satisfies the invariant. Increasing the space cannot result in a missing or duplicate movie or a negative number for available spaces. Thus, if it is broadcast and executed on another replica, it is sufficient that the pre-state satisfies the invariant to preserve it in the post-state.

### Definition 4 (Invariant-Sufficient)

A call *c* is invariant-sufficient iff for every state $\sigma$, if $\mathcal {I}(\sigma )$ then $\mathcal {P}(\sigma , c)$.

However, not all calls are invariant-sufficient. For example, a $\textsf {book}$ call may be permissible in a replica but may become impermissible in another when it is executed after an already executed $\textsf {offScreen}$ call for the same movie. These two calls should synchronize to preserve integrity. Nonetheless, some pairs of calls such as $\textsf {offScreen}$ and $\textsf {specialReserve}$ do not affect each other’s permissibility. (In the running example, $\textsf {specialReserve}$ has no guards. After an $\textsf {offScreen}$ call, it remains permissible as it doesn’t find the movie and leaves the relation unchanged).

### Definition 5 (Permissible-Right-Commutativity)

The call $c_1$ $\mathcal {P}$-R-commutes with the call $c_2$ written as $c_1 \rightarrow _\mathcal {P}c_2$ iff for every state $\sigma$, if $\mathcal {P}(\sigma , c_1)$ then $\mathcal {P}(\mathsf{update}(c_2)(\sigma ), c_1)$.

If a call $c_1$ is invariant-sufficient or $\mathcal {P}$-R-commutes another call $c_2$, then the call $c_1$ will stay permissible when it is propagated and applied to another replica even if $c_2$ is executed before it in that replica.

### Definition 6 (Permissible-Concur and Permissible-Conflict)

A call $c_1$ $\mathcal {P}$-concurs with a call $c_2$ iff $c_1$ is invariant-sufficient or $c_1 \rightarrow _\mathcal {P}c_2$. Otherwise, $c_1$ $\mathcal {P}$-conflicts with $c_2$.

The call $\textsf {offScreen}$ $\mathcal {P}$-concurs with the call $\textsf {specialReserve}$; however, the call $\textsf {book}$ $\mathcal {P}$-conflicts with the call $\textsf {offScreen}$.

We say that two calls *concur* iff they both $\mathcal {S}$-commute and $\mathcal {P}$-concur with each other. Otherwise, we say they *conflict* and need synchronization.

### Definition 7 (Concur and Conflict)

A pair of calls $c_1$ and $c_2$ concur iff they $\mathcal {S}$-commute and $\mathcal {P}$-concur with each other. Otherwise, they conflict $c_1 \bowtie c_2$.

**Dependency.** As we saw above, invariant-sufficient method calls can always preserve the invariant. However, there are calls whose preservation of the invariant is dependent on the calls that have executed before them at that replica. For example, taking the movie off-screen $\textsf {offScreen}$ is dependent on cancelling the last booking $\textsf {cancelBook}$. If $\textsf {offScreen}$ is moved left before $\textsf {cancelBook}$, it can become impermissible. Nonetheless, taking a movie off-screen $\textsf {offScreen}$ is independent of the previous special reservations $\textsf {specialReserve}$.

### Definition 8 (Permissible-Left-Commutative)

A call $c_2$ $\mathcal {P}$-L-commutes a call $c_1$, written as $c_2 \leftarrow _\mathcal {P}c_1$ iff for every $\sigma$, if $\mathcal {P}(\mathsf{{update}}(c_1)(\sigma ), c_2)$ then $\mathcal {P}(\sigma , c_2)$.

A call can avoid tracking dependencies to another call if the former is invariant-sufficient or $\mathcal {P}$-L-commutes with the latter.

### Definition 9 (Independent and Dependent)

A call $c_2$ is independent of $c_1$, written as , iff either $c_2$ is invariant-sufficient or $c_2 \leftarrow _\mathcal {P}c_1$. Otherwise, $c_2$ is dependent on $c_1$, written as .

If $c_1$ is executed before $c_2$ in the originating replica of $c_2$ and $c_2$ is dependent on $c_1$, then $c_2$ should be applied to other replicas only if $c_1$ is already applied.

**Recency.** Calls executed at a replica may be delayed in the network before they are executed in other replicas. Further, they may be buffered at the originating replica to reduce communication. The *pending* calls for a replica are the calls that have executed in other replicas but not at that replica yet. The staleness of a replica is the difference of its current state and its state after applying its pending calls. Given a bound $\epsilon$, a replica is sufficiently recent if its staleness is less than $\epsilon$. The calls that have originated in the current replica *n* but have not been received yet by another replica $n'$ make the state of $n'$ stale. To bound the staleness of $n'$ by $\epsilon$, the staleness imposed to $n'$ by the calls originated by each of the other $|\mathcal {N}| - 1$ replicas should be bounded by $\epsilon / (|\mathcal {N}| - 1)$. The difference that these calls can make is bounded by the sum of their weights (defined in Sect. 2). The staleness bound can be evenly divided between the replicas. However, in general it can be distributed unevenly and even dynamically. In particular, replicas that tend to issue updates more often can get a larger share.

Given a recency bound, a buffering quota can be calculated for each replica and the recency bound can be preserved when calls are buffered. Buffering calls can reduce communication; however, it can affect the convergence and integrity properties. To preserve these properties a buffer should have three properties: all-state-commutativity, invariant-sufficiency and let-$\mathcal {P}$-R-commutativity. We consider each condition in turn.

### Definition 10 (All-State-Commutative)

A call is all-$\mathcal {S}$-commutative if it is $\mathcal {S}$-commutative with respect to every call.

The calls of the buffer are executed locally and are not synchronized with other replicas. Therefore, if the buffer is not all-$\mathcal {S}$-commutative, concurrent execution of $\mathcal {S}$-conflicting calls in other replicas can lead to divergence. Similarly, if the buffer is not invariant-sufficient, concurrent execution of $\mathcal {P}$-conflicting calls in other replicas can lead to impermissibility of the buffer when it is propagated and executed in other replicas. The buffer in Fig. 3(c) is all-$\mathcal {S}$-commutative: it includes $\textsf {increaseSpace}$ and $\textsf {specialReserve}$ calls that result in increasing or decreasing the space for movies; the result is $\mathcal {S}$-commutative with respect to all method calls. Further, it is invariant-sufficient if the net result of its calls is a non-negative addition to the space of each movie. For example, if the $\textsf {increaseSpace}$ calls add *s* spaces and the $\textsf {specialReserve}$ calls subtract $s'$ spaces from the same movie where $s' \le s$, then the net effect is adding spaces and the buffer is invariant-sufficient.

### Definition 11 (Let-Permissible-Right-Commutative)

A call is let-$\mathcal {P}$-R-commutative if every call $\mathcal {P}$-R-commutes with it.

Calls in other replicas are checked to be permissible with no knowledge of the buffered calls in the current replica. Let-$\mathcal {P}$-R-commutativity of the buffer of the current replica guarantees that the calls in other replicas will continue to be permissible once they are propagated and executed after the buffer in the current replica. The buffer in Fig. 3(c) is let-$\mathcal {P}$-R-commutative; it may only increase the number of spaces that cannot make any call impermissible.

## Replicated System Semantics

In this section, we define the operational semantics of replicated objects where (1) the integrity property $\mathcal {I}$ on the state of each replica is always preserved, (2) replicas converge to the same state once all the calls are propagated, and (3) the staleness of each replica is always bounded by $\epsilon$. The semantics declares the conditions for execution and propagation of method calls on the replicated object to guarantee the three properties. In particular, it represents the conditions for local buffering of method calls to avoid communication while preserving the recency of the other replicas. In Sect. 5, we will see a static analysis that infers staleness bounds for the state. In this section, the semantics preserves the inferred staleness bound $\epsilon$ for the state $\sigma$ of the object. (For objects with multiple pieces of state, the staleness of each piece can be tracked separately.) The semantics strives to concisely define the conditions; we will present the protocols that implement these conditions in Sect. 6.

**Fig. 4.**
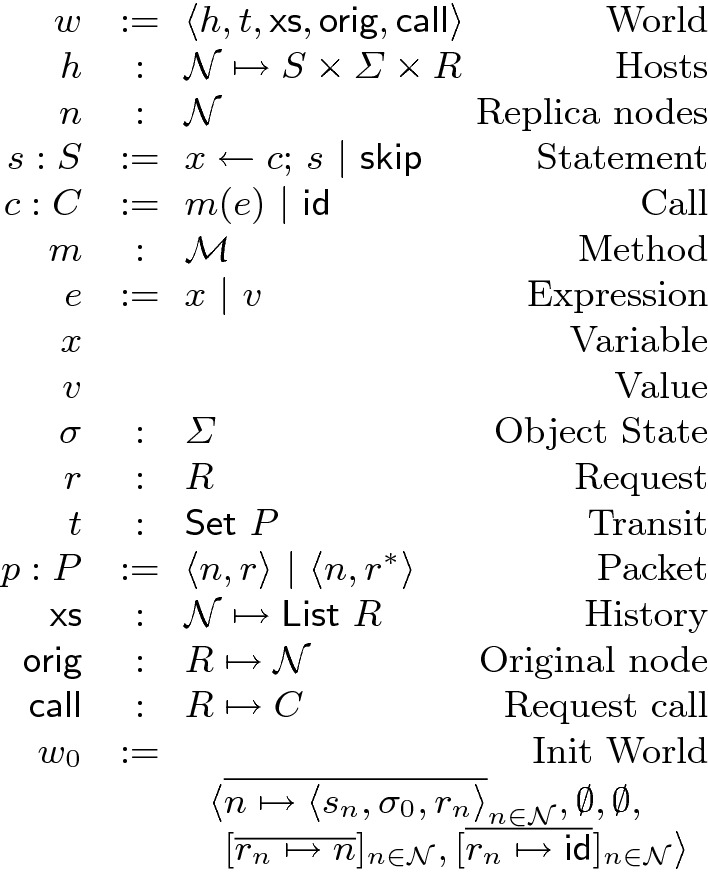
Operational semantics state

As Fig. 4 shows, the global state of the replicated system is represented as a world *w* that is a tuple of $\langle h, t, \textsf {xs}, \textsf {orig}, \textsf {call}\rangle$. The hosts *h* is a mapping from replica identifiers $\mathcal {N}$ to the local state of replicas. Each call is assigned a unique request identifier *r* at the originating replica. The two maps $\textsf {call}$ and $\textsf {orig}$ keep a mapping from request identifiers to the call and the originating replica of the request respectively. The state of each replica is a statement $s \in S$, the state of the object $\sigma \in \varSigma$, and the identifier $r \in R$ of the current buffer. A statement *s* is either $x \leftarrow c; s'$ that is the sequence of a call *c* and another statement $s'$, or the terminal statement $\mathsf {skip}$. A call *c* is the application of a method *m* to an argument expression *e*. A call can also be the identity call $\textsf {id}$ that leaves the state unchanged. (It is assumed that client statements do not make $\textsf {id}$ calls.) The network *t* is the set of packets that are sent but not yet delivered. A packet *p* contains the identifier of the destination replica *n* and the request identifier *r* of the call. If a packet is transmitting a buffered call, it is decorated with an asterisk $*$. The history $\textsf {xs}$ is a mapping from replica identifiers $\mathcal {N}$ to the list of request identifiers of the calls that are previously applied to that replica. The initial value of the world state is $w_{0}$ where each replica *n* hold its initial statement $s_n$, the initial state $\sigma _0$ of the object that satisfies the integrity property $\mathcal {I}$, and an empty buffer. Empty buffers are represented by mapping the buffer identifier $r_n$ of each replica *n* to the identity call $\textsf {id}$.

**Fig. 5.**
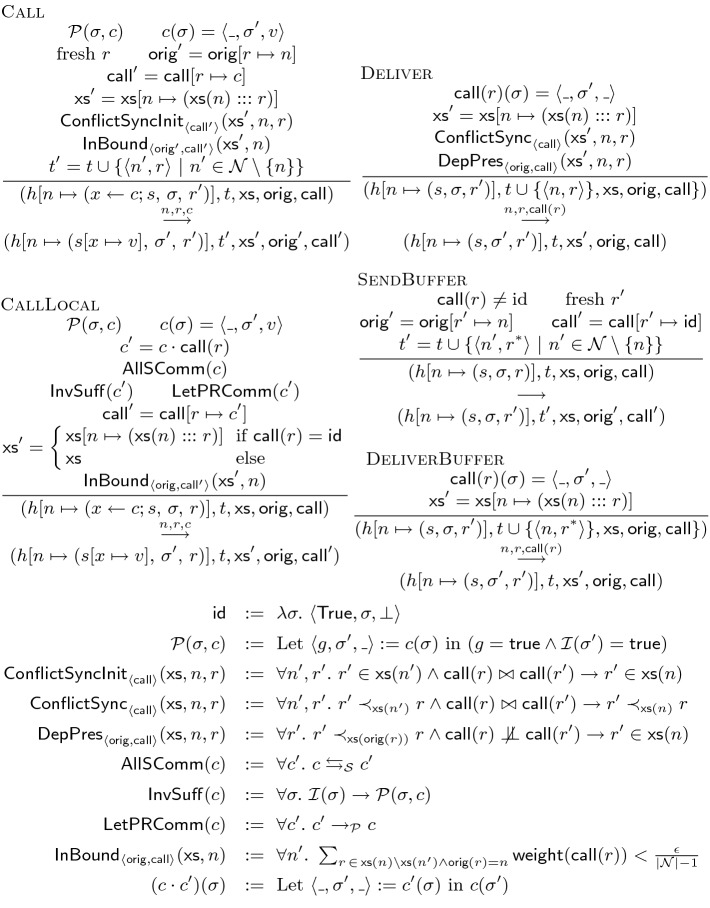
Replicated system semantics

Figure 5 presents the operational semantics. The rule Call executes a method call *c* at a replica *n*. The call *c* can be executed if the following conditions hold. (1) To preserve integrity, the call *c* should be locally permissible $\mathcal {P}(\sigma , c)$ in the current state $\sigma$. (2) To preserve convergence and integrity, any pair of conflicting calls should have the same order across the replicas, a property that we call conflict-synchronization. Thus, to execute a new request *r*, the rule Call requires the condition $\mathsf {ConflictSyncInit}$: any call $r'$ that is already executed in another replica $n'$ and conflicts with the current call *r* should have been already executed in the current replica *n*. Otherwise, once the calls *r* and $r'$ are propagated and executed on the other replicas, they will have different orders in the two replicas *n* and $n'$. (3) To preserve recency, this rule requires the condition $\mathsf {InBound}$: the difference that the pending calls from the current replica *n* can make to the state of every other replica $n'$ should be bounded by $\epsilon / (|\mathcal {N}| - 1)$. If the conditions above hold, a fresh identifier *r* is created for the call, the history $\textsf {xs}$ and the maps $\textsf {orig}$ and $\textsf {call}$ are updated to reflect the new call, a packet is sent in the network *t* to every other replica, and the variable *x* is substituted with the returned value *v* of the call in the continuation statement *s* of the current replica.

The rule Deliver delivers a call that has been sent to the current replica. It requires two conditions: conflict-synchronization and dependency-preservation. (1) Similar to the rule Call, conflict-synchronization requires $\mathsf {ConflictSync}$: if a conflicting call $r'$ is executed before the received call *r* in another replica $n'$, then $r'$ should have been already executed before *r* in *n* as well. (2) To preserve integrity, the dependencies of calls should be preserved. Thus, the dependency-preservation condition $\mathsf {DepPres}$ requires that a call *r* originated from a replica $n'$ is executed in the current replica *n* only if the calls $r'$ that have been executed before *r* in $n'$ and *r* is dependent on $r'$ should have been already executed in *n*.

Recency-aware replication can be applied to any object, but it can improve performance when there are method calls that can be buffered. The rule CallLocal executes a call but locally buffers it. Similar to the rule Call, it first checks the local permissibility of the call *c*. Since a buffered call is not immediately coordinated with calls in other replicas, it should satisfy the three properties (that saw in Sect. 5) to make it concur with any call: (1) all-state-commutativity $\mathsf {AllSComm}$, (2) invariant-sufficiency $\mathsf {InvSuff}$, and (3) let-$\mathcal {P}$-Right-commutativity $\mathsf {LetPRComm}$. The identifier of the current buffer is *r*; the current call *c* is composed with the current buffered call $\textsf {call}(r)$ to result in a composed call $c'$ for the updated buffer. The composition $\cdot$ of calls simply cascades their updates to the state. The all-state-commutativity condition is stated for single calls *c* (that implies the same condition for the composed call $c'$ as well). This condition is required for the call *c* because there might be other calls delivered between the last buffered call and the currently buffered call *c*. The call *c* should state-commute past the calls in between. Further, as explained for the rule Call, the condition $\mathsf {InBound}$ requires that the added staleness remains within bound. If the above conditions hold, the map $\textsf {call}$ is updated with the new buffer call $c'$, and the identifier *r* of the buffered call is added to the history $\textsf {xs}$, if the buffer was empty and the current call *c* is the first buffered call.

The rule SendBuffer sends the buffer to every other replica and resets the buffer. Packets transmitting buffers are decorated with an asterisk. The rule DeliverBuffer receives a packet containing a buffer. As we saw in the rule CallLocal, buffers are checked to be invariant-sufficient in the originating replica. Therefore, on receiving a packet containing a buffer, in contrast to the rule Deliver, the rule DeliverBuffer does not checks the dependency-preservation$\mathsf {DepPres}$ and the conflict-synchronization $\mathsf {ConflictSync}$ conditions.

The following lemmas state the three properties of the semantics. The following lemma states that once the buffers are flushed $\textsf {call}(r) = \textsf {call}(r') = \textsf {id}$ and the messages are delivered $t = \emptyset$, the replicas converge to the same state.

### Lemma 1 (Convergence)

For all *h*, *n*, $n'$, $\sigma$, $\sigma '$, *r* and $r'$, if $w_{0} \longrightarrow ^* \langle h, \emptyset , \_, \_, \_ \rangle$ where $h(n) = \langle \_, \sigma , r\rangle$, $h(n') = \langle \_, \sigma ', r'\rangle$ and $\textsf {call}(r) = \textsf {call}(r') = \textsf {id}$ then $\sigma = \sigma '$.

The following lemma states that every call enjoys the integrity property.

### Lemma 2 (Integrity)

For all *h*, *n*, *r*, *c*, *w* and $\sigma$, if $w_{0} \longrightarrow ^* \langle h, \_, \_, \_, \_ \rangle \overset{n, \_, c}{\longrightarrow } w$ where $h(n) = \langle \_, \sigma , \_\rangle$ then $\textsf {integrity}(\sigma , c)$.

The staleness of a replica is the difference of its current state and its state after applying its pending calls from others (buffered calls and in transit calls). The following lemma states that the stateless of every replica is bounded by $\epsilon$.

### Lemma 3 (Recency)

For all *h*, $h'$, *n*, *s*, $\sigma$ and $\sigma '$, if $w_{0} \longrightarrow ^*\langle h, \_, \_, \_, \_\rangle (\longrightarrow \cup \overset{n, \_, \_}{\longrightarrow })^* \ \langle h', \_, \_, \_, \_ \rangle$,$h(n) {\,=\,} \langle s, \sigma , \_ \rangle$, and $h'(n) {\,=\,} \langle s, \sigma ', \_ \rangle$ then $\varDelta (\sigma ', \sigma ) < \epsilon$.

## Staleness Bound Inference and Optimization

In Sect. 4, we presented an operational semantics that preserves a given staleness bound for the state. The users declare the recency that they expect from the return value of each method of the object. The specified bounds for the methods can be used to infer the bounds for the elements of the state. In this section, given an object specification that includes recency declarations for the methods, we present a static analysis that infers optimum staleness bounds for each element of the state. We present a syntax-directed analysis that derives recency constraints between bound variables for the state elements. A solution to the constraints assigns a bound value to each state element such that if every state element keeps its staleness bound then the result of every method call respects the recency declaration of the method. The optimum solution maximizes the (weighted) sum of the bounds to increase buffered calls and hence decrease communication.

**Fig. 6.**
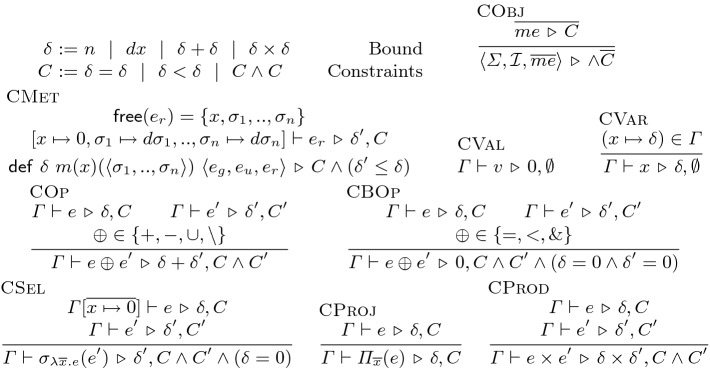
Bound constraint derivation

Figure 6 presents the constraint inference rules for the object language that we saw in Fig. 1. A delta bound $\delta$ is either a natural number *n*, a delta variable *dx*, or addition or multiplication of two deltas. A constraint *C* is equality or comparison of two deltas, or conjunction of two constraints. A delta environment $\Gamma$ is a mapping from variables to delta variables or values. The judgements are of the following forms: the judgement $o \, \triangleright \, C$ states the bounding constraint *C* for the object *o*, the judgement $m \, \triangleright \, C$ states the constraint *C* for the method *m*, and the judgement $\Gamma \vdash e \, \triangleright \, \delta , C$ states that under the delta environment $\Gamma$, the staleness of the expression *e* is bounded by $\delta$ when the constraints *C* are satisfied. The rule CObj states that the constraint for an object is the conjunction of the constraints for its methods. (We assume that the state variables passed to all the methods are renamed to the same variables $\langle \sigma _1, .., \sigma _n\rangle$.) The rule CMet infers the constraints for a method by first, inferring the constraints for its return expression under a delta environment where the argument is mapped to the delta value of zero (exactly recent) and the state variables $\sigma _i$ are mapped to delta variables $d \sigma _i$ to be inferred, and second, bounding the return value. The rule CVal assigns the delta value zero to values with no constraints. (Values are exact.) The rule CVar retrieves the bindings for delta variables from the environment. The rule COp states that the delta for the result of the operators $\{+, -, \cup , \setminus \}$ is the sum of the delta of its operands. On the other hand, the rule CBOp requires the operands of the boolean operators $\{=, <,  \&  \}$ to be exact and states that the result is exact as well. We elide the similar rule for the unary negation operator !. The rule CSel requires the selection condition to be exact and states that the delta of the resulting relation is the same as the input relation. In other words, the resulting relation is stale by the same number of elements as the input relation. Similarly, the rule CProj states that the delta of the resulting relation is the same as the input relation. On the other hand, the rule CProd states that the delta for the resulting relation is the multiplication of the deltas for the input relations. In our running example, let us associate the bound variables *drs* and *dms* to *rs* and *ms* respectively. The constraint inferred for $\textsf {querySpace}$ is $dms \le \epsilon _2$, for $\textsf {queryReservations}$ is $drs \le \epsilon _1$, and for $\textsf {querySpace}$ that involves the join operator (product and selection) is $drs \times dms \le \epsilon _3$. More detailed explanation for these derivation is available in the appendix [5].

We now define the notion of sufficiently-recent states. Intuitively, a state is sufficiently-recent with respect to the target state if the difference of the return value of every method call on that state versus the target state is within the declared bound of the method.

### Definition 12 (Sufficiently-recent State)

A state $\langle v_1, .., v_n\rangle$ is a sufficiently-recent state with respect to the target state $\langle v_1^*, .., v_n^*\rangle$ for an object *o* iff for every method $\mathsf {def} \ \epsilon \ m(x)(\langle \sigma _1, .., \sigma _n\rangle ) \ \langle e_g, e_u, e_r\rangle$ of *o*, and every argument *v*, let $v_r$ be  and $v_r^*$ be , we have $\varDelta (v_r, v_r^*) \le \epsilon$.

The following lemma states that the bound inference presented in Fig. 6 is sound. In other words, if the inference derives the constraints *C* for an object, for any solution *S* of *C*, if the staleness of each state element $\sigma _i$ of the object remains within the bound $S(d\sigma _i)$, then the state remains sufficiently-recent.

### Lemma 4 (Soundness of Bound Inference)

Given an object *o* with the state variables $\langle \sigma _1, .., \sigma _n\rangle$, if $o \, \triangleright \, C$ that is the constraints *C* (over the bound variables $\overline{d \sigma _i}$) are derived for *o*, and *S* is a solution for *C*, then for every pair of states $\sigma = \langle v_1, .., v_n\rangle$ and $\sigma ^* = \langle v_1^*, .., v_n^*\rangle$, if $\overline{\varDelta (v_i, v_i^*) < S(d \sigma _i)}$ then $\sigma$ is sufficiently-recent for $\sigma ^*$.

There may be many solutions for the derived constraints, and hence, many sound state bounds that preserve the user-specified bounds for the object. However, solutions that allow more staleness (albeit appropriately bounded) are more favorable since they allow more buffered calls and require less communication. Thus, a candidate objective function to maximize is $d\sigma _1 + .. + d\sigma _n$. In other words, what are the largest delta bounds for the state elements that still preserve the recency specifications of the methods? This function gives the same weight to all the state elements; however, some may be updated more frequently. Let $f_i$ be the relative update frequency of the state element $\sigma _i$. Frequencies can be obtained from historical logs or profiling. The objective function is defined as the following weighted sum $d\sigma _1 / f_1 + .. + d\sigma _n / f_n$. More frequently updated state elements are given proportionally larger bounds. In our running example, let $\epsilon _1 = 3$, $\epsilon _2 = 4$, and $\epsilon _3 = 6$. If the update frequency of *rs* is twice as *ms*, the optimum solution is $drs = 3$ and $dms = 2$.

### Definition 13 (Recency Bound Optimization)

Give an object *o* and the relative update frequency $f_i$ of the state elements $\sigma _i$ of *o*, if $o \, \triangleright \, C$ then the optimum staleness bounds for *o* are the solution *S* of *C* that maximizes $\textsf {d}\sigma _1 / f_1 + .. + d\sigma _n / f_n$.

It is obvious that the objective function can be easily translated to a linear function by multiplying the least common denominator of the frequencies.

## The Power and the Protocol of Recency-Aware Objects

Now, we show that recency-aware objects are stronger than the perfect failure detector abstraction [18] and present a protocol that implements recency-aware objects using perfect failure detectors. These two results show that recency-aware objects have the same computational power as the perfect failure detector.

The perfect failure detector abstraction $\mathcal {P}$ notifies processes about the crash of the other processes in a synchronous network. It has the following properties: Liveness: Every crashed process is eventually detected by all correct processes. Safety: No correct process is ever suspected by other processes. The recency-aware object $\mathcal {R}$ has the following liveness and safety properties. Liveness: If the user makes a request to a correct replica, it eventually responds. Safety: Executed calls that are yet pending for each correct replica is bounded. The following lemma states that $\mathcal {P}$ is reducible to $\mathcal {R}$ and also its opposite, $\mathcal {R}$ is reducible to $\mathcal {P}$.

**Fig. 7.**
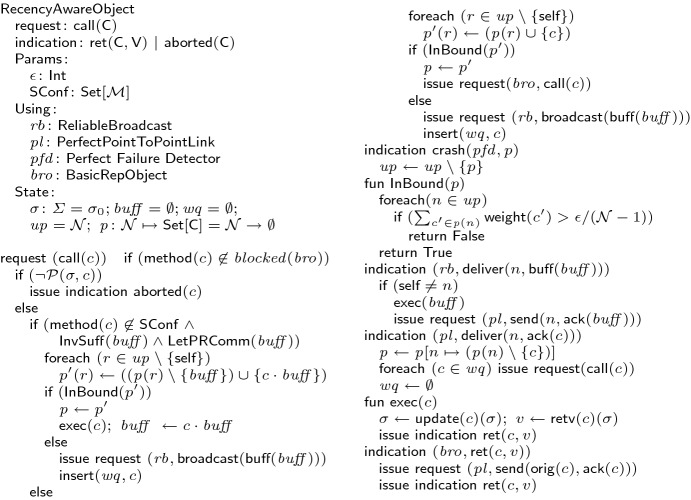
Recency-aware protocol

### Lemma 5

$$\mathcal {P} \preceq \mathcal {R} \ \wedge \ \mathcal {R} \preceq \mathcal {P}$$.

For the proof of the first conjunct, consider two replicas $$ rep _1$$ and $$ rep _2$$. We show by contradiction that $$ rep _1$$ will eventually know whether $$ rep _2$$ has crashed. We assume the opposite. Consider an execution where $$ rep _1$$ has already executed a set of requests *R* and receives another request *r* from the user, such that the pending set $$R \cup \{r\}$$ makes a difference in the state of $$ rep _2$$ that pushes it out-of-bound. By the contradiction assumption, $$ rep _1$$ is never informed when $$ rep _2$$ crashes. Therefore, if $$ rep _1$$ does not hear from $$ rep _2$$, the following two scenarios are indistinguishable to $$ rep _1$$. ($$S_1$$) The replica $$ rep _2$$ has crashed. ($$S_2$$) The replica $$ rep _2$$ is too slow. The replica $$ rep _1$$ has the following two choices: ($$C_1$$) The replica $$ rep _1$$ waits to hear from $$rep_2$$ about receiving a request in *R* before processing and responding to *r*. ($$C_2$$) The replica $$ rep _1$$ processes and responds to *r*. If the protocol makes the choice $$C_1$$, it might be the scenario $$S_1$$ and then the liveness property is violated. If the protocol makes the choice $$C_2$$, it might be the scenario $$S_2$$ and then the recency bound for $$ rep _2$$ is violated.

The second conjunct, directly follows from the protocol. We briefly describe the protocol in Fig. 7 that implements a recency-aware replicated object. The full description of the protocol is available in the appendix [5]. Given an object definition, the protocol benefits from both static and dynamic coordination analysis to guarantee convergence, integrity and recency. To reduce communication, replicas try to execute the calls locally while maintaining the staleness bound $$\epsilon $$. Each replica keeps its locally executed calls in a buffer $$ buff $$ before they are broadcast. Replicas send an acknowledgement $$\textsf {ack}$$ to the originating replica once they receive and execute a call or a buffer of calls. Each replica $$ rep $$ keeps a map called pending *p* from each replica $$ rep '$$ to the set of pending calls sent from $$ rep $$ to $$ rep '$$. When a replica originates a call *c*, it adds *c* to its local pending set for each of the other replicas; once it receives an acknowledgement for *c* from a replica $$ rep' $$, it removes *c* from the set of pending calls for $$ rep' $$. Each replica keeps the set of correct replicas $$ up $$, and removes a replica from the set if the prefect failure detector $$ pfd $$ issues a crash event for that replica. A requested call can be executed only if it does not push the pending set for any correct replica out of the bound. Otherwise, it cannot be immediately executed and is kept in a waiting queue $$ wq $$ to be retried later, and further, the buffer is sent to the other replicas and is reset to accelerate the shrinking of the pending set. To decide whether a call can be executed locally, the conditions of the rule CallLocal of the operational semantics (Sect. 4) are checked. The set of state-conflicting methods $$\textsf {SConf}$$ that is statically calculated is consulted to check if the call is all-state-commutative. The validity of the two conditions invariant-sufficiency and let-$$\mathcal {P}$$-R-commutativity of the buffer (after the new call is added) are dynamically decided by a solver at run-time. If the conditions do not hold, the call is coordinated with other replicas using the basic blocking coordination protocol $$ bro $$ [30] that guarantees integrity and convergence but not recency.

**Fig. 8.**
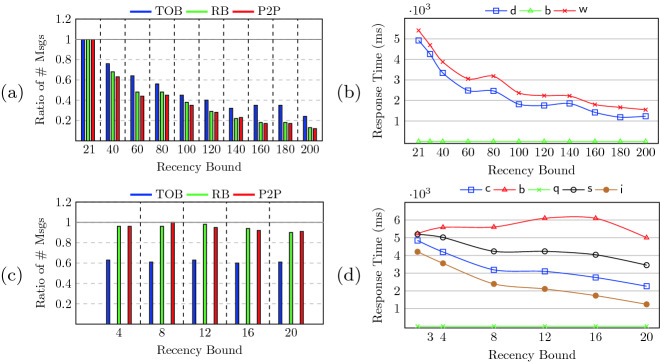
Effect of recency on coordination load and response time. (a) and (b) show the bank account use-case. $\mathsf {d}$, $\mathsf {w}$, and $\mathsf {b}$ stand for $\mathsf {deposit}$, $\mathsf {withdraw}$ and $\mathsf {balance}$ (with the frequencies of 75%, 25%, 5% in the workload respectively). (c) and (d) show the movie use-case. $\mathsf {c}$, $\mathsf {b}$, $\mathsf {q}$, $\mathsf {s}$, and $\mathsf {i}$ stand for $\mathsf {cancelBook}$, $\mathsf {book}$, $\mathsf {querySpace}$, $\mathsf {specialReserve}$, and $\mathsf {increaseSpace}$ (with frequencies 4%, 6%, 5%, 40% and 45%).

## Experimental Results

We have implemented the analysis and protocol as a synthesis tool called $\textsc {Hampa}$. We applied it to two use-cases: the bank account use-case (with the $\textsf {withdraw}$, $\textsf {deposit}$ and $\textsf {balance}$ methods and the integrity property of non-negative balance) and the movie booking use-case (Fig. 2). The experiments show that as the staleness bound increases, the coordination overhead and response time of recency-aware objects is decreased. Further, recency-aware objects are twice as responsive as sequentially consistent counterparts.

**Platform and Setup.** The experiments are conducted on a cluster of 4 computing nodes. Each node has 2 AMD Opteron 6272 CPUs with a total 8 cores, 64GB ECC memory and 40Gbps InfiniBand network. JDK is openjdk version 1.8.0_222. We used the CVC4 [11] SMT solver v.1.7. Reported numbers are the arithmetic means of results from three repetitions on 4 replicas. In the experiments for the bank account use-case, all the calls are applied to the same account object and the amount is selected randomly in the range [10, 20]. For the movie use-case, we send requests for each movie identifier to the same replica. Further, we do not issue $\textsf {offScreen}$ calls because taking a movie off-screen causes later method calls on the same movie to be aborted and thus, these methods are not fully exercised. This would significantly improve the response time. However, in practice, $\textsf {offScreen}$ calls are rarely used. The movie and user IDs are chosen at random from six and a hundred unique IDs. In all the experiments, we execute 500 calls in millisecond intervals evenly distributed between 4 replicas.

**Measurements.** We measure two comparison criteria: coordination load and response time. At the lower layers, the protocol reduces to three communication primitives: total-order-broadcast (TOB), reliable-broadcast (RB) and point-to-point links (P2P). To measure the coordination overhead, we separately count the number of different types of messages that replicas send during the execution of their requests. The response time for a call is the duration between the time that the client requests the call and the time that the user receives the return value.

We performed three experiments. In the first experiment, we study the effect of increasing the staleness bound on the coordination load. We report the ratio of the number of messages that the protocol sends for the bound under test over the number of messages that it sends for the base-line bound. (The base-line recency bound is the maximum weight of the calls. The baseline allows every single call to be buffered.) In the second experiment, we study the effect of increasing the staleness bound on the response time of each method. Finally, in the last experiment, we compare the response time of our protocol with the base-line recency, with the sequential consistency (SC). SC uses total-order broadcast for all the methods.

**Assessment.** Figure 8(a) and (c) show the effect of increasing the staleness bound on the coordination load for the two use-cases. As the staleness bound is increased, the ratio of the messages sent by RB, TOB and P2P decreases. Figure 8(a) (bank account), shows 88% decrease in the number of messages sent to RB when the bound is increased from 20 to 200. Likewise, the TOB and P2P ratios decrease by 78% and 90%, respectively. In Fig. 8(c) (movie booking), buffering helps to reduce TOB calls by 40% across the experiments. This decrease, however, unlike the bank account use-case, is steady over different bounds. This is because it is more difficult to “buffer” in the movie booking use-case. There are no $\mathcal {S}$-conflicts in the bank account use-case and hence two out of two update methods can be buffered. However, $\mathcal {S}$-conflicts in the movie use-case allow only 2 out of 4 update methods to be buffered: $\textsf {increaseSpace}$ and $\textsf {specialReserve}$. Also, we observe that the number of RB and P2P messages decrease by at most 10%.

**Fig. 9.**
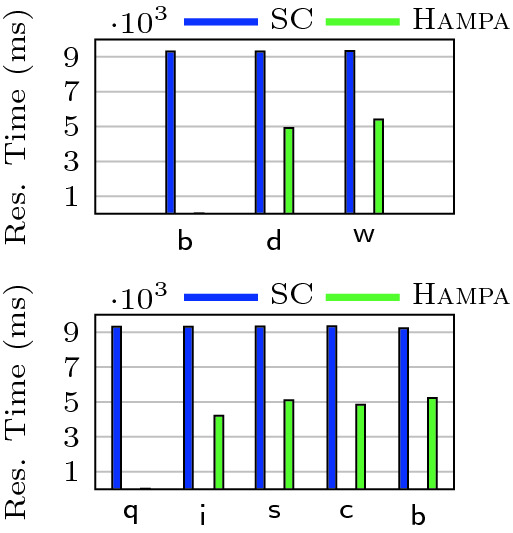
Response time comparison between $\textsc {Hampa}$ and sequential consistency for each method type. Top: bank account, Bottom: movie booking use-case.

Figure 8(b) and (d) shows the effect of increasing the staleness bound on the response time for the two use-cases. In Fig. 8(b) (bank account), the response time of $\textsf {withdraw}$ and $\textsf {deposit}$ methods decrease by 71% and 75%, respectively when the staleness bound is increased from 20 to 200. The $\textsf {withdraw}$ method is the least responsive method. The reason is that it has a self-conflict and requires synchronization if it cannot be buffered. In Fig. 8(d) (movie booking), we observe slight increase in response time for the $\textsf {book}$ method while increasing the bound from 2 to 20. This is because the $\textsf {book}$ operation cannot be buffered due to the $\mathcal {S}$-conflict with other methods and has to be synchronized. On the other hand, the response time of the $\textsf {specialReserve}$ method decreases by 33% when the bound is increased from 2 to 20. The reason is that it has a self-conflict and if it cannot be buffered, it should be synchronized by the TOB and TOB incurs a high coordination overhead. Therefore, as buffered calls increase and the use of TOB decreases, the response time is significantly improved. The response time of the $\textsf {increaseSpace}$ method also benefits from recency awareness; it decreases by 72%. The methods $\textsf {book}$ and $\textsf {cancelBook}$ have conflicts. In the blocking protocol that $\textsc {Hampa}$ uses, the method $\textsf {book}$ handles synchronization; therefore, the method $\textsf {cancelBook}$ just broadcasts the request. As the recency bound is increased, the network is less crowded and therefore, the response time of $\textsf {cancelBook}$ is decreased.

Figure 9 compares the response time of recency-aware objects with the baseline bound with the sequentially consistent objects. The $\mathsf {SC}$ protocol synchronizes all the calls and orders them with respect to each other. However, $\textsc {Hampa}$ minimizes coordination while preserving convergence, integrity and recency. We observe that the response time speedup is in average as high as $2\times$ and $1.8\times$ for the bank account and movie use-cases respectively. More experiments are available in the appendix [5]. In particular, they show that the runtime cost of SMT solving is only 0.2% to 1% of the average response time.

## Related Work

Epsilon serializability [46] allows concurrent execution of updates with queries and bounds the difference of the inconsistent values that are observed in these executions and the consistent values that would be observed in a serializable execution. In contrast, $\textsc {Hampa}$ preserves the integrity of the state, bounds staleness, allows different orders in different replicas, and formally defines the difference for relational operators.

In TACT [54–58], operations return tentative values; they might be eventually reordered to preserve strong consistency. TACT bounds the numeric error between the tentative and final return values. The user specifies the granularity of the bounded object “conit” and the strength of the protocol. On the other hand in $\textsc {Hampa}$, the states are final and enjoy integrity provided on top of weak consistency. Further, the staleness bound with respect to the pending future state is automatically optimized with static and dynamic analyses.

In AQuA [31], given a query and a staleness bound, the master server dynamically selects a recent enough server to service the query. Similarly, TRAPP [43] finds recent enough servers for different parts of data that are needed for the query. FRACS [59] allows operations to be buffered at replicas up to a given threshold. In contrast to $\textsc {Hampa}$, these projects do not guarantee integrity and convergence, and do not automatically infer the staleness bounds. PIQL [6] bounds the number of key-value store operations for each query trading the precision of the result for performance. However, it does not consider the staleness of replicas.

To reduce synchronization, PBS [9] communicates with only a partial quorum of replicas to bring a total order to operations, and probabilistically bounds the staleness of the observed states. In contrast, $\textsc {Hampa}$ performs synchronization with full quorums but only for conflicting calls, and allows different orders for replicas. Further, it analyzes and synthesizes replicated objects and supports relational in addition to single-key operations.

The trade-off between consistency and latency presented as PACELC [1] aligns with our experiments. As the consistency decreases (staleness bound increases), the latency decreases (responsiveness increases). Warranties [38] and Homeostasis [47] allow local updates if they keep the validity of certain assertions. Although other replicas can rely on the validity of the assertions, the staleness of their state is not bounded. In contrast, $\textsc {Hampa}$ maintains a staleness bound. Further, it exploits weak consistency and guarantees convergence.

## Conclusion

This paper presented a relational object specification language that captures the integrity and recency requirements of the object. It presented a syntax-directed analysis that given a specification, infers optimum staleness bounds. In addition, it presented the coordination avoidance conditions, operational semantics, a protocol and a synthesis tool for replicated systems that guarantee convergence, integrity and recency. The recency-aware protocol embeds a solver to decide whether coordination avoidance is safe and increases the responsiveness.
